# Genome-Wide Identification and Expression Analyses of *Glycoside Hydrolase Family 18* Genes During Nodule Symbiosis in *Glycine max*

**DOI:** 10.3390/ijms26041649

**Published:** 2025-02-14

**Authors:** Rujie Li, Chuanjie Gou, Ke Zhang, Milan He, Lanxin Li, Fanjiang Kong, Zhihui Sun, Huan Liu

**Affiliations:** Guangdong Provincial Key Laboratory of Plant Adaptation and Molecular Design, Innovative Center of Molecular Genetics and Evolution, School of Life Sciences, Guangzhou University, Guangzhou 510006, China

**Keywords:** soybean, *glycoside hydrolase family 18*, class V chitinase, nodulation

## Abstract

Glycoside hydrolase family 18 (GH18) proteins can hydrolyze the β-1,4-glycosidic bonds of chitin, which is a common structure component of insect exoskeletons and fungal cell walls. In this study, 36 *GH18* genes were identified and subjected to bioinformatic analysis based on the genomic data of *Glycine max*. They were distributed in 16 out of 20 tested soybean chromosomes. According to the amino acid sequences, they can be further divided into five subclades. Class III chitinases (22 members) and class V chitinases (6 members) are the major two subclades. The amino acid size of soybean GH18 proteins ranges from 173 amino acids (aa) to 820 aa and the molecular weight ranges from 19.46 kDa to 91.01 kDa. From an evolutionary perspective, soybean *GH18* genes are closely related to Medicago (17 collinear loci with soybean) and Lotus (23 collinear loci with soybean). Promoter analysis revealed that *GH18* genes could be induced by environmental stress, hormones, and embryo development. *GmGH18-15*, *GmGH18-24,* and *GmGH18-33* were screened out due to their nodulation specific expression and further verified by RT-qPCR. These results provide an elaborate reference for the further characterization of specific *GH18* genes, especially during nodule formation in soybean.

## 1. Introduction

Chitin is the second most abundant biomolecule in nature, only led by cellulose. Chitin or chitin oligosaccharides (chitooligosaccharides) are the polymer or oligomer of N-acetylglucosamine (GlcNAc), which is linked by a β-1,4-glycosidic bond [1]. In most cases, chitin is the component of the exoskeleton of arthropods and the cell walls of fungi. Chitinases are capable of cleaving β-1,4-glycosidic bonds and thus releasing GlcNAc residues with different degrees of polymerization [2]. Distinguished by the structure of catalytic domains, chitinases can be primarily grouped into two categories, namely glycoside hydrolase family 18 and 19 (GH18 and 19). The classic feature of GH18 members is a barrel-like structure consisting of eight α-helices and eight β-sheets, while GH19 members have a lysozyme-like structure with several α-helices, which is specifically found in plants [3]. Based on amino acid sequence similarity, chitinases can also be classified into five groups, with class III and V belonging to GH18 and class I, II, and IV belonging to GH19 [4].

The active center of GH18 proteins contains a conserved module, (DN)-X-(DN)-X-E, which is crucial for their enzymatic activities [5]. Except for the catalytic domain, class III and V chitinases are quite different from each other. Class III chitinases do not have a carbohydrate-binding module (CBM), while class V chitinases often contains two CBMs and a c-terminal extension which is responsible for the vacuolar localization [1].

There is no chitin production in plants, which leads the biological function of plant chitinases to the interaction with environment cues or microbes. The chitinase activities could be induced in soybean roots by the ethylene treatments and beneficial microbes [6,7]. *AtChiA*, a class III chitinase gene in *Arabidopsis thaliana*, was specifically up-regulated in response to salt stress and wounding [8]. Class V chitinases are also considered as pathogen-related protein family 11 (PR-11) proteins [9,10]. For example, NtChiV, the first class V chitinase, was purified from *Nicotiana tabacum* when the leaves were inoculated with the tobacco mosaic virus, which exhibited antifungal activity [11,12]. AtChiC, a class V chitinase from *A. thaliana*, was reported to be induced by abscisic acid, jasmonic acid, flagellin, NaCl, and osmosis [13].

Besides the involvement in abiotic and biotic stress, GH18 proteins could also play a role in the interaction between plants and their symbionts. Through the establishment of symbiosis with mycorrhizal fungi and rhizobia, plants can take more P and N, respectively [14]. The expression levels of the tested class I, II, and IV chitinase genes were not changed in mycorrhizal roots of Medicago; however, there were three class III chitinase genes which were strongly up-regulated [15]. Similarly, *QrchitIII-1*, a class III chitinase gene from *Quercus robur*, was up-regulated in lateral roots during the pre-mycorrhization stage with *Piloderma croceum* [16]. The inoculation of the grapevine with the arbuscular mycorrhizal fungus *Glomus versiforme* induced the expression of class III chitinase gene *VCH3* (*Vitis amurensis* class III chitinase) [17]. Class III chitinases also might take part in the nodule symbiosis. *Srchi13* was considered as an early nodulin gene due to its specific and transient expression during nodule formation in *Sesbania rostrata*. The recombinant Srchi13 possessed the lipochitooligosaccharide degradation capacity [18], while in *Casuarina glauca*, *Cgchi3* was specifically indued in actinorhizal nodules [19]. Unlike class III chitinases, the biological contribution of class V chitinase to the plant–microbe interaction is mainly limited to nodule symbiosis. In Medicago, *MtNFH1* (*Nod factor hydrolase 1*) and *MtCHIT5b* (*class V chitinase b*) were not only inducible by rhizobial inoculation or Nod factor treatment, but also their gene products could degrade the Nod factors from *Sinorhizobium meliloti* with high substrate affinity. Nodule formation is strongly influenced in their gene mutants, exhibiting abnormal nodule branching and reduced nodule number, respectively [20,21,22,23]. In Lotus, the deficiency of *LjCHIT5* (class V chitinase) also caused the abnormal elongation of infection threads within the nodule cortex and later caused mature nodule formation [24].

Soybean (*Glycine max* (L.) Merr.) is the most important leguminous crop. It is the largest source of food and animal feed protein and the second largest source of vegetable oil in the world [25]. Taking full advantage of nodule symbiosis is an environmentally friendly approach for sustainable agriculture with less chemical fertilizer investment. Although there are a few publications focused on the biological relevance of soybean chitinases in pathogen invasion and drought stress conditions [26,27], the role of soybean chitinases during nodule development is severely overlooked.

In this study, our primary objective is to better understand the soybean *GH18* genes, particularly their relevance in nodule symbiosis. Therefore, a comprehensive bioinformatic analysis was performed with the *GH18* genes in soybean, including chromosomal positioning, conserved motifs/domains, the phylogenetic relationship with other legumes, and expression profile, especially in nodulation. These results provide valuable resources for the further gene function characterization of soybean *GH18* genes.

## 2. Results

### 2.1. Chromosomal Distribution of G. max GH18 Genes

**Figure 1:**
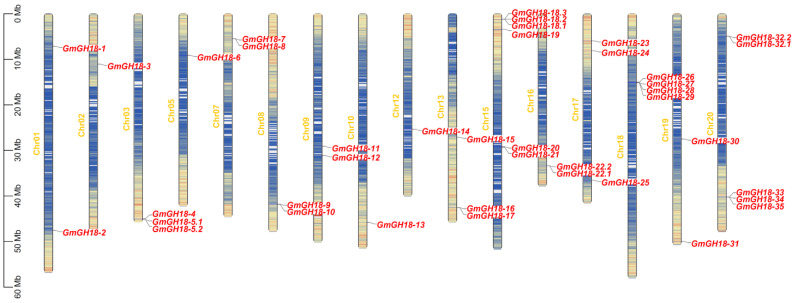
Chromosome mapping of the *GH18* genes in *G. max*. Here, 20 soybean chromosomes were displayed in scale with a ruler beside. The position of each *GH18* gene was clearly marked on the chromosomes. The yellow to blue gradient represents different gene density, with yellow as high gene density region and blue as low gene density region.

According to the Pfam code (PF00704 for glycoside hydrolase family 18 catalytic domain) and HMM search result of soybean protein sequences (download from https://data.jgi.doe.gov/refine-download/phytozome?organism=Gmax, accessed on 10 December 2024), a total of 36 putative *GH18* genes were identified (Table 1). Among them, Glyma.03G256800, Glyma.15G015100, Glyma.16G173000, and Glyma.20G035400 possessed multiple versions of transcripts. These genes were widely distributed in the soybean genome, which are mapped to 16 chromosomes (Figure 1). Chromosome 04, 06, 11, and 14 do not possess any *GH18* genes. There are four *GH18* genes on chromosomes 15, 18, and 20, respectively, while three *GH18* genes were on chromosomes 13 and 17. The other chromosomes have 1–2 *GH18* genes on them. Due to the integrity of Wm82.a2.v1 genome assembly, Glyma.U033800.1 had not been mapped to any chromosome by this point, but later turned out to be on chromosome 11 in Wm82.a4.v1.

### 2.2. Analysis of Phylogenetic Tree, Conserved Motifs and Conserved Domains

**Figure 2:**
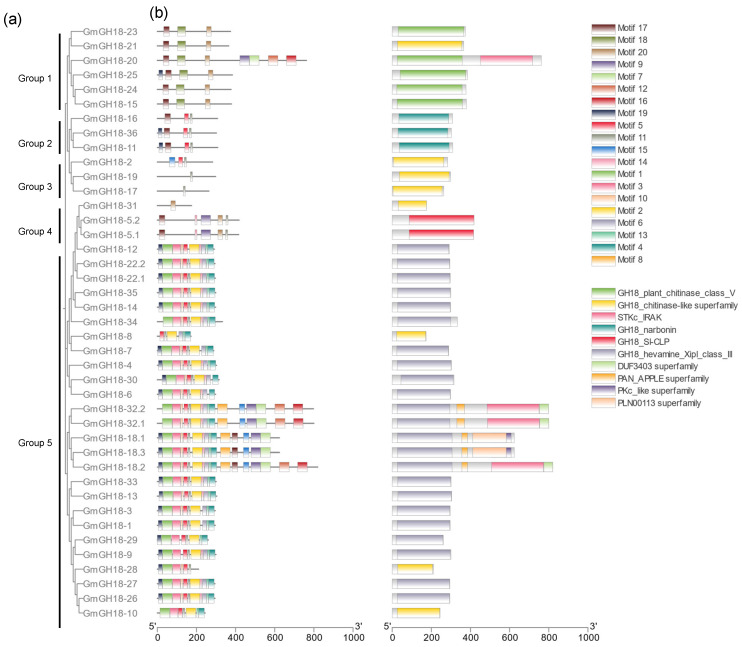
Phylogenetic analysis and protein structure identification of *GH18* genes in soybean. (**a**) A phylogenetic tree was constructed by the Maximum Likelihood method. Bootstrap tests with 1000 replicates were performed. (**b**) Here, 10 conserved motifs and 10 conserved domains were identified in soybean GH18 proteins, respectively.

Based on the deduced amino acid sequences from Phytozome v13, a phylogenetic tree was constructed for soybean GH18 family members. Meanwhile, motif analysis and conserved domain identification were performed. Accordingly, 36 putative *GH18* genes could be classified into 5 groups, namely class V chitinase, narbonin, chitinase-like superfamily, stabilin-1 interacting chitinase-like protein (SI-CLP), and xylanase inhibitor/class III chitinase (Figure 2). Narbonin contains a barrel-like structure, so it is considered to belong to GH18 [28]. The majority of GH18 proteins (22 out of 36) belong to the class III chitinase clade which showed a high homology degree. Motifs 19, 1, 3, 10, 5, 11, 2, 14, 6, 13, and 4 are frequently shown in this clade (see motif sequences in Figure S1). Motif 8 is exclusively detected in GmGH18-18 and GmGH18-32, which is correlated with the PAN/apple domain. The combination of motifs 15, 9, and 7 is another feature for GmGH18-18 and GmGH18-32, which is correlated with the serine/threonine kinase domain. There are two kinds of kinase domain involved, both STKc_IRAK and PLN00113, whose main difference is the presence of motif 12 and 16. The combination of motifs 17, 18, and 20 is the signature of class V chitinase (6 out of 36). Noteworthy, GmGH18-20 is the only class V chitinase which contains a serine/threonine kinase domain in group 1. There are three, three, and two GH18 proteins in group 2, 3, and 4, respectively. To some extent, GH18 proteins from group 2, 3, and 4 are similar to class V chitinase. However, motifs 17, 18, and 20 are not completely present in their protein sequences.

**Figure 3:**
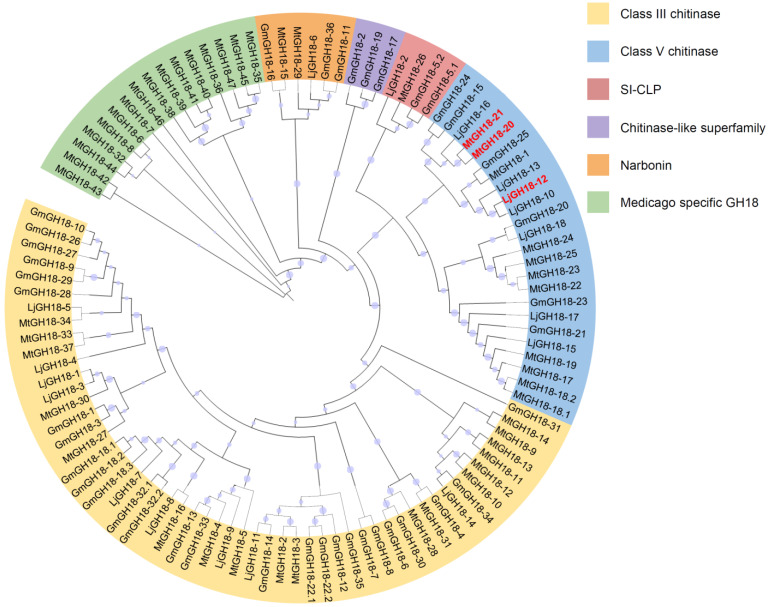
Phylogenetic analysis of GH18 proteins in soybean, Medicago, and Lotus. Based on the HMM search result of PF00704, 36, 47, and 18 putative GH18 proteins were identified in soybean, Medicago, and Lotus, respectively. They were further divided into 6 groups using the Maximum Likelihood method implemented on MEGA7.0. There were 50 genes in the class III chitinase clade, 23 genes in the class V chitinase clade, 3 genes in the stabilin-1 interacting chitinase-like protein clade (SI-CLP), 3 genes in the chitinase-like superfamily, 6 genes in the narbonin clade, and 16 genes in the Medicago-specific GH18 clade, respectively. Due to the reported biological function in legume–rhizobium symbiosis, MtGH18-20 (MtNFH1), MtGH18-21 (MtCHIT5b), and LjGH18-12 (LjChit5) are highlighted in red.

In order to have a revolutionary perspective on the significance of GH18 proteins, a more complicated phylogenetic tree was constructed, involving soybean, Medicago, and Lotus (see Medicago and Lotus *GH18* gene IDs in Table S1) (Figure 3). These species are legumes, which can establish symbiosis with rhizobia. The biological function of several GH18 proteins from Medicago and Lotus have been thoroughly characterized during nodulation [18,19,20,21,22,23,24]. Nearly all GH18 proteins could be categorized into the five clades used for soybean GH18 classification; however, there are quite a lot of special GH18 proteins from Medicago (16 out of 47 Medicago GH18) that could not fit in. Therefore, they are uniformly named as Medicago-specific GH18. Class III chitinase remain to be the biggest clade of GH18, in which there are 18 and 9 (out of 18) members from Medicago and Lotus, respectively. In the class V chitinase clade, there are 11 proteins from Medicago and 7 proteins from Lotus. Noteworthy, *GmGH18-15* and *GmGH18-24* are most closely related to *MtGH18-20* (*MtNFH1*) and *MtGH18-21* (*MtCHIT5b*), while *GmGH18-25* is most closely related to *LjGH18-12* (*LjChit5*), indicating that these soybean counterparts might also play a role in nodule symbiosis [22,23,24]. In the SI-CLP clade, each contains 1 GH18 member for Medicago and Lotus. In the chitinase-like superfamily, there are no homologous proteins for Medicago and Lotus. In the narbonin clade, there are two Medicago GH18 proteins and one Lotus protein. Taken together, the GH18 proteins show more or less the same evolutionary trend among the three modern legume species.

### 2.3. Physicochemical Property Analysis of GH18 Proteins in G. max

To better understand the protein properties of soybean GH18 proteins, molecular size, isoelectric point, protein stability and hydropathicity were studied in detail. As shown in Table 1, soybean GH18 proteins have a wide range of amino acid size, from 173 aa (GmGH18-8) to 820 aa (GmGH18-18.2), which is mainly due to the presence of the C-terminal serine/threonine kinase. The average amino acid length is 368. Thus, the molecular weight ranges from 19.46 kDa (GmGH18-8) to 91.01 kDa (GmGH18-18.2), and the average molecular weight is 40.38 kDa. The pI ranges from 4.07 to 9.42, among which 22 GH18 proteins have a pI lower than 7.0 and the remaining 19 GH18 proteins have a pI higher than 7.0. The ratio of acidic enzymes to basic enzymes is about 1:1. As for the instability, the majority of soybean GH18 proteins have a value below 40, which is considered stable. Those from the SI-CLP clade, especially for GmGH18-5.1 and GmGH18-5.2, are unstable. The Aliphatic index of soybean GH18 proteins range from 70.99 (GmGH18-23) to 92.02 (GmGH18-20), which indicates the wide variation in thermal stability. The grand average of the hydropathicity of soybean GH18 proteins is basically negative, ranging from −0.352 (GmGH18-17) to 0.000 (GmGH18-2).

Plants cannot produce chitin, but the chitinases could be used to degrade exogenous chitin. Thus, whether the given chitinase can be secreted out of the cell after protein synthesize is a precondition for its biological function. To a large extent, the presence of a signal peptide can determinate its secretion. Signal peptide prediction was performed for soybean GH18 proteins. Most of them have a functional secretion signal, except for GmGH18-7, GmGH18-8, and those from the SI-CLP clade and chitinase-like superfamily.

### 2.4. Collinearity Analysis of GH18 Genes in Soybean, Medicago, and Lotus

**Figure 4:**
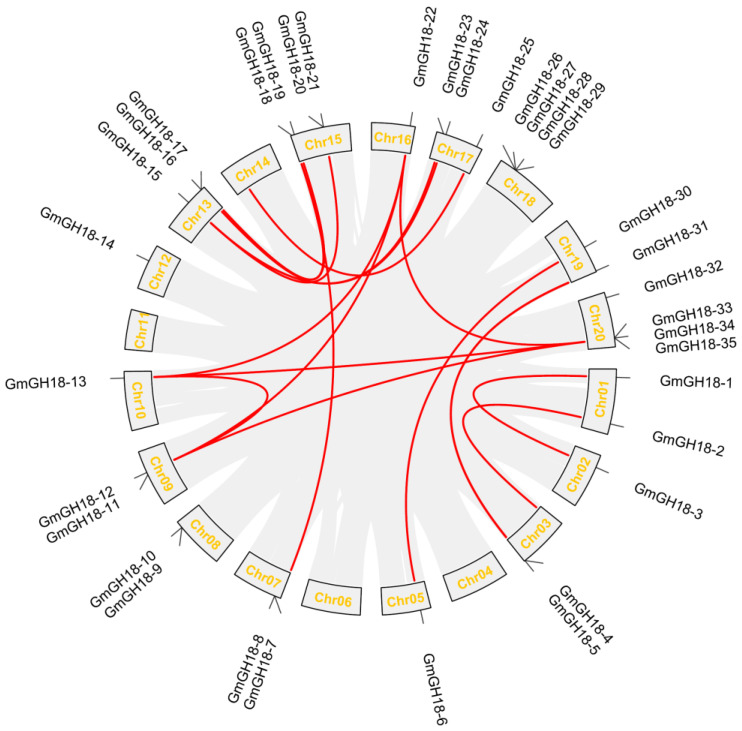
Collinearity analysis of *GH18* genes in soybean. Here, 20 soybean chromosomes were arranged in circle. Gray lines represent the gene pairs in the whole genome. Red lines highlight the collinear relationship within the *GH18* gene family.

Soybean has undergone whole genome duplication twice during evolution, which allows plants to acquire new genes and create genetic novelty [29,30]. An analysis of correlated gene arrangements could facilitate deducing a common ancestor [31]. In soybean, 19 pairs of fragment copy genes were identified in the *GH18* gene family (Figure 4 and Table S2). Chromosome 13 has the most fragment replication genes (6 pairs), followed by chromosome 15, with four pairs of fragment replication genes. Chromosomes 03, 09, 10, 16, 17, 19, and 20 each have three pairs of fragment replication genes. Chromosome 01 has two pairs of fragment replication genes. Chromosomes 02, 05, 07, and 14 each have one pair of fragment replication genes. In contrast, there were none in chromosomes 04, 06, 08, 11, 12, and 18. Pairs of genes within the same chromosomes were not detected. In short, the *GH18* gene pairs identified above could be a good implication for soybean whole genome duplication.

**Figure 5:**
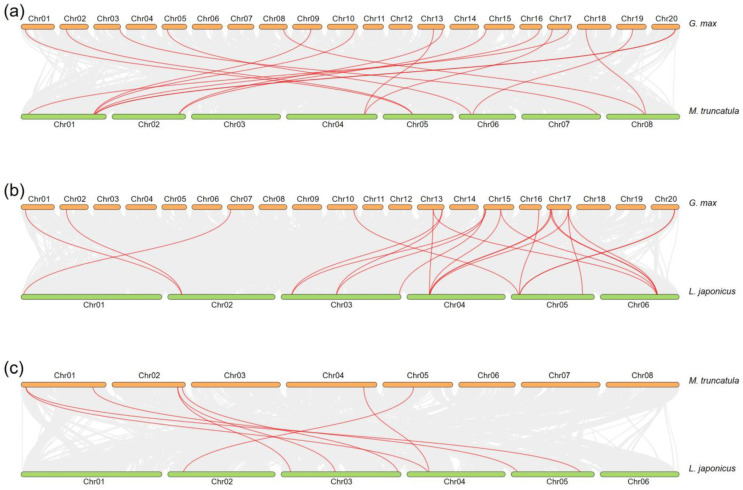
Collinearity analysis of *GH18* genes between two species of soybean, Medicago, and Lotus. Collinearity analysis was performed between two given species: soybean–Medicago (**a**), soybean–Lotus (**b**), and Medicago–Lotus (**c**). Gray lines from the background show all collinear gene pairs between two genomes, while red lines highlight the collinear relationship within the GH18 family.

To obtain a larger evolutionary profile of *GH18* genes in legumes, collinearity analysis was conducted between two species of soybean, Medicago, or Lotus. As shown in Figure 5a and Table S3, there are 17 soybean *GH18* genes that have a common ancestor with Medicago. Chromosomes 13, 17, and 20 have the most gene pairs, each with two gene pairs. Five out of twenty soybean chromosomes have no gene pairs with Medicago. Furthermore, there are in total 23 soybean *GH18* genes which have a source relationship with Lotus, namely 7 homologous gene pairs in chromosome 17, 5 homologous gene pairs in chromosome 15, 4 homologous gene pairs in chromosome 13, 2 homologous gene pairs in chromosome 20, and 1 homologous gene pair in chromosomes 01, 02, 07, 10, and 16 (Figure 5b and Table S4). Only 9 out of the 20 soybean chromosomes have gene pairs with Lotus. Collinearity analysis was then performed between Medicago and Lotus, whose genome sizes are much smaller than soybean. The results showed that there are only eight gene pairs, among which three were in Medicago chromosome 02 and Lotus chromosome 03 (Figure 5c and Table S5).

### 2.5. Analysis of Cis-Acting Elements in G. max GH18 Gene Promoter Regions

**Figure 6:**
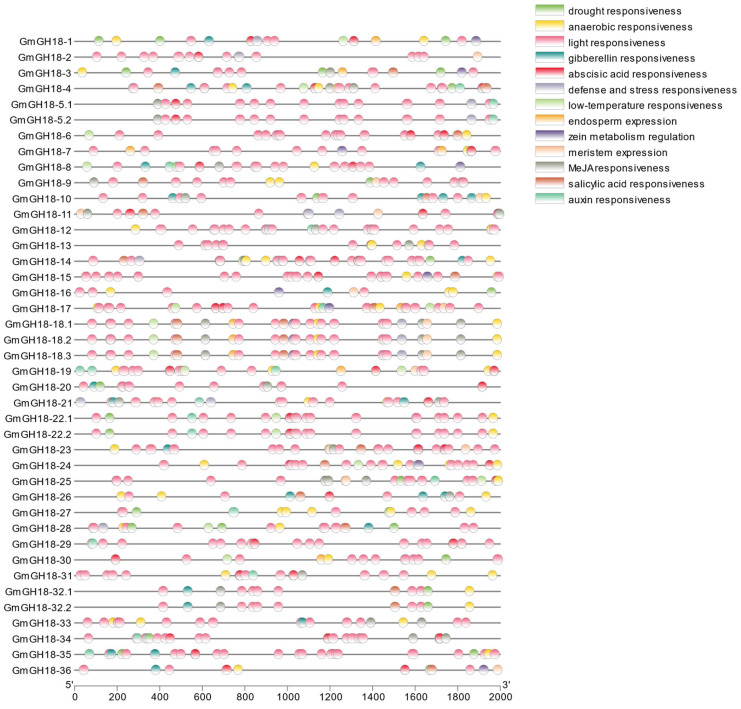
Cis-acting element analysis of *GH18* gene promoter regions in *G. max*. 41 *GH18* gene promoter sequences (−2000 bp upstream of ATG) are obtained from *G. max* Wm82 genome (a2.v1). The gene symbols are listed on the left and the nucleotide positions are labeled at the bottom. In total, 13 cis-acting elements are identified, whose potential biological functions are related to drought responsiveness, anaerobic responsiveness, light responsiveness, gibberellin responsiveness, abscisic acid responsiveness, defense/stress responsiveness, low-temperature responsiveness, endosperm expression, zein metabolism regulation, meristem expression, Methyl jasmonate (MeJA) responsiveness, salicylic acid responsiveness, and auxin responsiveness.

For a better understanding of the biological function of soybean *GH18* genes, the cis-acting elements in their promoter regions were identified. Both abiotic stress (e.g., anaerobic responsiveness) and biotic stress (e.g., defense/stress responsiveness) conditions are included. Positions of such cis-acting elements are labeled in the region of 2000 bp upstream of the translation initiation site (Figure 6).

Light responsive elements were identified in all GH18 family members in soybean, among which *GmGH18-14* contained the most elements, with up to 20. Methyl jasmonate-responsive elements were identified in 21 *G. max* GH18 family members, among which *GmGH18-11*, *GmGH18-18*, and *GmGH18-25* contained the most elements, with 6. Abscisic acid-responsive elements were identified in 29 *G. max* GH18 family members, with *GmGH18-34* containing 7. Anaerobic-responsive elements were identified in 31 *G. max* GH18 family members, among which *GmGH18-27* contained the most elements, with 5. Drought-responsive elements were identified in 19 *G. max* GH18 family members, among which there are 1–3 elements, respectively. Gibberellin-responsive elements were identified in 16 *G. max* GH18 family members, among which *GmGH18-10* contained the most elements, with 4. Salicylic acid-responsive elements were identified in 16 *G. max* GH18 family members, among which there are 1–2 elements for each gene. Auxin-responsive elements were identified in 15 *G. max* GH18 family members, among which *GmGH18-19* contained the most elements, with 3. Defense/stress-responsive elements were identified in 11 *G. max* GH18 family members, among which *GmGH18-12* contained the most elements, with 3. Low-temperature-responsive elements were identified in twelve *G. max* GH18 family members, among which the majority of them contained only one element. Meristem expression-related elements were identified in 11 *G. max* GH18 family members, among which there are 1–2 elements for each gene. Zein metabolism regulation-related elements were identified in 11 *G. max* GH18 family members, among which all genes contained only one element. Endosperm expression-related elements were identified in eight *G. max* GH18 family members, among which all genes contained only one element, except for *GmGH18-7,* with two elements.

Furthermore, the transcription binding sites were also analyzed to predict the potential regulators of the *G. max GH18* genes. As shown in Figure S2, there are a number of TFBSs in the 2000 bp promoter regions. AT-Hook, TBP, Homeodomain, Myb/SANT, WRKY, bHLH, bZIP, and TCR were mainly identified due to higher frequencies. AT-Hook, TBP, Homeodomain, Myb/SANT, bZIP, and TCR were identified in all *G. max GH18* gene promoter regions, with 3513, 2377, 1885, 1680, 740, and 628, respectively. There were 1159 WRKY that were identified in 37 *G. max GH18* gene promoter sequences. There were 836 bHLH that were identified in 39 *G. max GH18* gene promoter sequences.

### 2.6. The Expression of GH18 Genes During Nodule Symbiosis

**Figure 7:**
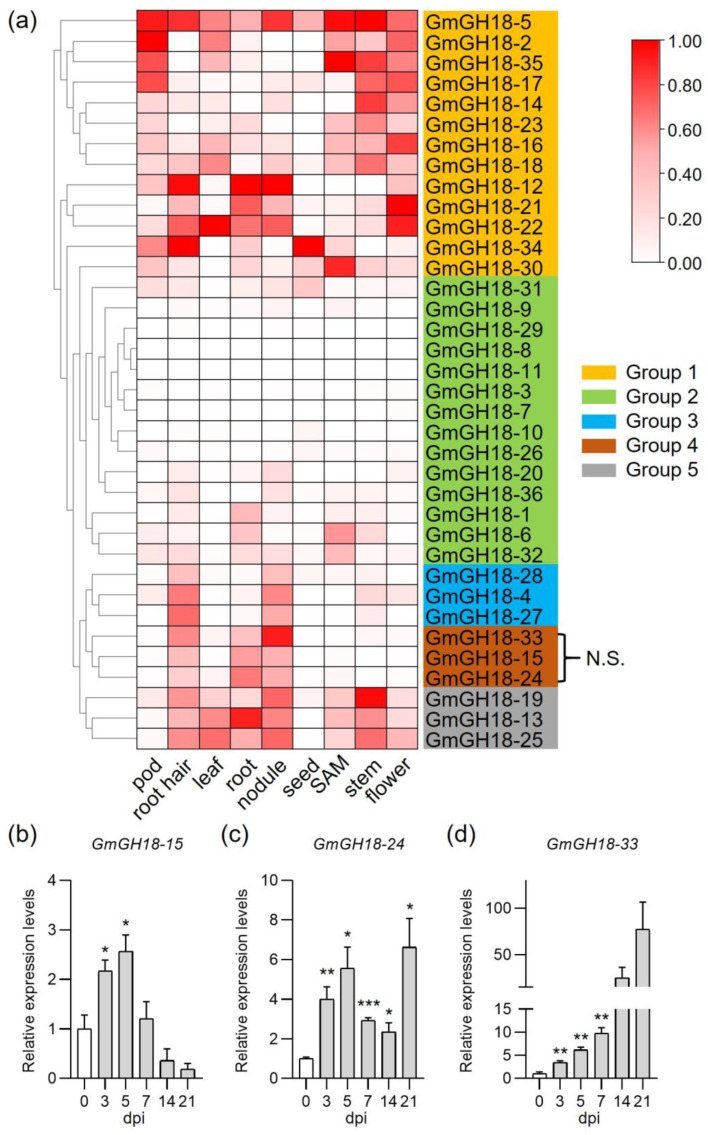
Expression profile of soybean *GH18* genes and rhizobial responsiveness validation by RT-qPCR. (**a**) The *GH18* gene expression pattern was extracted from the soybean RNA sequencing data [32] and displayed in the heatmap. According to their expression levels in pods, root hairs, leaves, roots, nodules, seeds, shoot apical meristems (SAM), stems, and flowers, 36 soybean *GH18* genes were grouped into 5 categories. Group 4 was highlighted, because members from this group are nodulation-specific (N.S.). (**b**–**d**) RT-qPCR validation of the expression behavior of group 4 members under symbiotic conditions. *G. max* Wm82 plants were inoculated with *Bradyrhizobium japonicum* USDA110; the transcript levels of *GmGH18-15*, *GmGH18-24*, and *GmGH18-33* in the roots (within 7 days post inoculation) and nodules (14 and 21 dpi) were measured (*n* = 3). The *Actin* gene served as a reference. Data indicate means ± SE of normalized expression values (mean value of control set to one). The asterisks indicate significantly increased expression compared to control roots without rhizobial inoculation (Student’s *t*-test; * *p* < 0.05, ** *p* < 0.01, *** *p* < 0.001).

To better understand their biological functions, the expression profile of soybean *GH18* genes was investigated in detail. According to the different expression patterns, they can be classified into five groups (Figure 7a). Those from group 1 generally exhibit ubiquitous expression from the whole plant. *GmGH18-5* shows a high expression level in all tested tissues. In contrast, members from group 2 showed extremely low expression in all tissues. Members from group 3, 4, and 5 have low expression in pods and seeds but have relative high expression in root hairs and nodules. Those from group 5 also have higher expression in shoots, like leaves, shoot apical meristem (SAM), stem, and flower. The difference between group 3 and 4 is the expression in root. Those from group 3 show low expression in root, while those from group 4 show the opposite.

Through the root system, legumes can acquire nitrogen during the interaction with rhizobia. Genes with higher expression in roots, root hairs, and nodules tend to play a role in nodule symbiosis. In other words, group 4 genes are very likely to be nodulation-related. To test this hypothesis, soybean roots (as well as nodules) inoculated with rhizobia were harvested for gene expression measurement at different time points. As shown in Figure 7b–d, the expression levels of *GmGH18-15*, *GmGH18-24*, and *GmGH18-33* can be induced by rhizobial inoculation. The expression of *GmGH18-15* decreased in nodules, while *GmGH18-24* and *GmGH18-33* remained highly up-regulated in nodules. Such results indicated that members from group 4 show good nodulation responsiveness and might be important for the establishment of symbiosis.

Since *GmGH18-15* and *GmGH18-24* are class V chitinase genes, the expression levels of other class V chitinase genes were further measured. *GmGH18-20*, *GmGH18-21*, *GmGH18-23*, and *GmGH18-25* showed no rhizobial induction at the early infection stage (Figure S3). Except for *GmGH18-23*, others showed lower expression in nodules. Thus, rhizobial responsiveness is a unique feature for group 4 members. Furthermore, the expression patterns of *GH18* genes in Medicago and Lotus during symbiosis were analyzed. The criteria includes (i) nodulation-related tissue specific expression and (ii) rhizobial induction at the early infection stage. The nodulation-specific members were highlighted. There are nine Medicago and two Lotus *GH18* genes classified into this group, respectively (Figures S4 and S5).

## 3. Discussion

In this study, 36 *GH18* genes were identified from the whole genome of soybean. Compared with a recent study about soybean chitinases, which claims that there are 19 class III chitinases and 6 class V chitinases, 11 more GH18 members have been newly found. Regarding class III chitinases, GmGH18-8, GmGH18-28, and GmGH18-29 are the new ones. Motif analysis clearly showed that they contained similar motifs to other class III chitinases (Figure 2). GH18 proteins from SI-CLP, the chitinase-like superfamily, and narbonin are firstly characterized. They do not possess too many similar motifs with class III or V chitinases, but they also contain chitinase-related domains (Figure 2). It is not clear whether they still display chitinase activity or whether they lost it during evolution. The classification of GmGH18-31 is ambiguous due to its low sequence homology with others. It is grouped together with GmGH18-5.1 and GmGH18-5.2 in the motif analysis (Figure 2), while it is also classified into class III chitinase in the phylogenetic analysis (Figure 3).

The genome of soybean (1.1 Gb) is larger than the genomes of Medicago (520 Mb) and Lotus (470 Mb). During evolution, soybean has undergone large-scale whole genome duplication twice, while Medicago and Lotus have experienced duplication before speciation [33]. Their genomes share a substantial collinearity (Figure 5). The homologous genes can be found in each sub-clade of *GH18*, with the exception that Medicago has its own specific *GH18*, which is worth further investigation. One benefit of gene duplication is the specialized neofunctionalization. *GmGH18-15* and *GmGH18-24* are a pair of fragment copy genes (Figure 4 and Figure S2) and both are inducible by rhizobia (Figure 7). Nevertheless, the expression levels of *GmGH18-15* are much reduced in nodules, while *GmGH18-24* remains highly expressed (Figure 7).

In some cases, plant chitinases are expressed at low levels and they can be specifically induced under certain conditions [34,35]. As shown in the cis-acting element analysis, environmental stress such as drought, anaerobic condition, light, abscisic acid, and cold temperature could influence their gene expression (Figure 6). Meanwhile, chitinases can also be induced by certain hormones such as gibberellin, methyl jasmonate, salicylic acid, and auxin (Figure 6) [13]. Certain biological progresses, such as embryo and meristem development, also need the expression of chitinases [36]. Furthermore, chitinases are widely considered as responsive to the microbe-associated molecular pattern, especially the cell wall components of fungi such as chitin, chitin oligosaccharides, and pectin polysaccharides. The recognition of these molecules by the corresponding receptors located on the plasma membrane initiates the signaling, and eventually, the expression of chitinases becomes a classic marker for plant defense. The synthesized chitinases can be secreted and further inhibit the growth of fungi by the hydrolysis of their cell walls. In agriculture, active chitinases are often used as biocontrol agents in the form of either purified molecules or plant gene transformation.

Legumes can obtain more nitrogen through the establishment of a symbiotic relationship with rhizobia. Nod factors, produced by rhizobia, are signal molecules, which can trigger the nodulation signaling in host legumes. Nod factors are modified chitin oligosaccharide consisting of at least a sugar backbone and an *N*-linked fatty acid chain at the non-reducing end [37]. The hydrolysis of Nod factors by host hydrolases has been studied for the past thirty years. Originally, the Nod factor degradation was found in *Vicia sativa*, *Medicago sativa*, *M. truncatula*, and pea [38,39,40]. Later, the specific enzymes were identified and characterized in *M. truncatula* and *L. japonicus*, which turned out to be class V chitinases [20,22,23,24]. Not only were these enzymes able to cleave Nod factors both in vitro and in vivo, but also the mutants deficient in these genes showed a strong nodulation phenotype. One biological significance of such hydrolysis is the inactivation of the nodulation signaling and the avoidance of overrated nodulation, which will cost a lot of energies. To date, the Nod factor hydrolases in soybean have not been identified yet. Interestingly, HgCht2, a homolog of plant class V chitinase from the soybean cyst nematode *Heterodera glycines*, was reported to be able to hydrolyze the Nod factors from both *S. meliloti* and *B. japonicum USDA110* and play a role in antagonizing the colonization of beneficial symbionts [41]. Therefore, it is quite possible that there are certain class V chitinases in soybean that are capable of degrading Nod factors. Indeed, *GmGH18-15* and *GmGH18-24* are the homologous genes of *MtNFH1* and *MtCHIT5b* in soybean (Figure 3). *GH18* gene collinearity analysis between soybean and Medicago showed that both *GmGH18-15* and *GmGH18-24* had common ancestors with *MtNFH1* (Figure 5, Table S3). Their expressions are inducible by rhizobia (Figure 7). On the other hand, the scenario in soybean could be similar with Lotus. *GmGH18-25* is closely related to *LjChit5*, *LjGH18-10*, and *LjGH18-13* (Figure 3). *LjGH18-10* and *LjGH18-13* have evolved as pseudogenes [24]. Synteny analysis revealed that *GmGH18-25* and *LjGH18-13* have common ancestors (Figure 5, Table S4). Although *GmGH18-25* can barely be up-regulated by rhizobia, it might play a role in nodule symbiosis (Figure 5).

In conclusion, our informatic analysis with soybean *GH18* genes provides a full picture of their chromosomal localizations, motif/domain structures, evolutionary cues, and expression patterns. These data are crucial references for the further characterization of specific *GH18* genes in soybean.

## 4. Materials and Methods

### 4.1. Sequence Acquisition

The whole genome sequences, protein sequences, and gene annotations of *G. max* (Wm82.a2.v1), *M. truncatula* (Mt4.0v1), and *L. japonicus* (Lj1.0v1) were obtained from the Phytozome server (https://phytozome-next.jgi.doe.gov/) [32,42,43]. In order to identify *GH18* genes in these three species, the catalytic domain of glycoside hydrolase family 18 (PF00704; http://pfam.xfam.org/) was used as a query code to search for in their protein sequences using the HMM Search function of TBtools-II (v2.119) [44]. The obtained hit rankings by both sequence scores and domain scores were taken as an intersection, and those with an E-value lower than 1 × 10^−8^ were considered as candidate *GH18* genes. Then, their genomic sequences and protein sequences were further extracted using Fasta Extract function of TBtools-II.

### 4.2. Analysis of Chromosomal Localization

According to the soybean GFF3 file, the chromosome mapping of *GH18* genes was performed using the Gene Localization Visualize function of TBtools-II.

### 4.3. Construction of Phylogenetic Tree

For the phylogenetic analysis of either soybean *GH18* genes or all *GH18* genes in three species, their full-length protein sequences were aligned firstly using Clustalx2.1 program, which was further used to construct a phylogenetic tree. The evolutionary history was inferred using the Maximum Likelihood method based on the Whelan And Goldman model [45]. Evolutionary analyses were conducted in MEGA7 with 1000 bootstrap tests [46]. Eventually, the phylogenetic tree was beautified with ITOL server (https://itol.embl.de/, accessed on 10 December 2024).

### 4.4. Identification of Conserved Motifs and Domains

MEME (https://meme-suite.org/meme/tools/meme, accessed on 10 December 2024) was used to identify 20 conserved motifs with other default settings in soybean GH18 protein sequences [47]. Conserved domains of soybean GH18 proteins were examined in the Conserved Domain Database of NCBI (https://www.ncbi.nlm.nih.gov/Structure/cdd/wrpsb.cgi, accessed on 10 December 2024) [48]. Finally, TBtools-II was used for the integration of the soybean GH18 evolutionary tree, conserved motifs, and conserved domains.

### 4.5. Analysis of Physicochemical Properties

Amino acids size, molecular weight, isoelectric point, instability index, aliphatic index, and grand average of hydropathicity were analyzed using the ProtParam online server (https://web.expasy.org/protparam/, accessed on 10 December 2024) [49]. The signal peptide was predicted using the SignalP-6.0 server (https://services.healthtech.dtu.dk/services/SignalP-6.0/, accessed on 10 December 2024) [50].

### 4.6. Collinearity Analysis

First, the soybean genomic sequences were analyzed by the Fasta Stats tool of TBtools-II to calculate the length of each chromosome. Next, the gene annotation file was processed by the Gene Density Profile tool of TBtools-II to obtain the gene density. Then, the Blast Compare Two Seqs tool was used to blast the soybean genomic sequences with themselves. Gene positions were acquired by the GFF3/GTF Gene Position (Info.) Parse tool. Lastly, the Advanced Circos tool was used to ornamented the result. Meanwhile, synteny analyses between two species were conducted in a similar way [51].

### 4.7. Analysis of Cis-Acting Elements and Transcription Factor Binding Sites

The 2000 bp sequences upstream of the start codon of soybean *GH18* genes were obtained using the Gtf/Gff3 Sequences Extract function of TBtools-II. Later, these sequences were submitted to the PlantCARE website to obtain the predicted cis-acting elements (http://bioinformatics.psb.ugent.be/webtools/plantcare/html/, accessed on 10 December 2024) [52]. The selected cis-acting elements were visualized using the Simple BioSequence Viewer function of TBtools-II. Similarly, the 2000 bp promoter sequences were uploaded to the PlantPan 4.0 online server to scan for the transcription factor binding sites (http://plantpan.itps.ncku.edu.tw/plantpan4/index.html, accessed on 10 December 2024) [53].

### 4.8. Gene Expression Analysis and Validation by Quantitative Real Time PCR

The expression data of *GH18* genes were downloaded from Phytozome for soybean, Medicago, and the Lotus Base (https://lotus.au.dk/, accessed on 10 December 2024) [54]. Due to an updated version of genome assembly used in the Lotus Base, Lotus *GH18* genes were searched for using a new gene ID by sequence blast, and then the corresponding expression data were extracted. The transcript levels were analyzed in different tissues under various conditions. The value was further normalized in the form of log2 and displayed in the heatmap.

For the validation experiments, *G. max* Wm82 seedlings were germinated in the vermiculite for 5 days and later inoculated with *B. japonicum* USDA110 (OD_600_ = 0.1 mL, 30 mL/plant). Roots were harvested at 0, 3, 5, and 7 days post inoculation, while nodules were harvested at 2 and 3 weeks post inoculation. RNA was extracted and used for gene expression analysis. The plant RNA purification kit and cDNA reverse transcription kit were purchased from Magen and Vazyme, respectively. The primers used are listed in Table S6. The expression of the *Actin* gene served as a reference. All statistical analyses were performed in the GraphPad Prism Statistical analysis and graphing software (Version 8.0.2). The means and significant *P* values were calculated using Student’s *t*-test.

## 5. Conclusions

As a key crop in global agriculture, soybean provides abundant plant-based protein for human beings. Biological nitrogen fixation is both an environmental-friendly and efficient approach for soybean to acquire nitrogen. Here, we performed bioinformatic analysis regarding *GH18* gene chromosomal localizations, motif/domain structures, and evolutionary cues and highlighted their inducibility during nodulation. These findings implicate that certain GH18 proteins play a role in nodule symbiosis. Selecting and fully utilizing the best GH18 protein(s) may improve soybean nodulation efficiency. Thus, this can reduce the input of chemical fertilizer and enlarge the nitrogen acquisition, leading to increased production with higher nitrogen content. Future research should focus on characterizing the GH18 enzymatic activities towards Nod factors, which will facilitate the understanding of the Nod factor signaling between rhizobia and legumes.

## Figures and Tables

**Table 1:** Physicochemical property analysis of GH18 proteins in *G. max*.

Gene Symbol	Gene ID	Amino Acids Size	Molecular Weight (kDa)	Isoelectric Point	Instability Index	Aliphatic Index	Grand Average of Hydropathicity	Signal Peptide
*GmGH18-1*	Glyma.01G055200.1	296	31.74	5.39	35.54	83.48	−0.113	Yes
*GmGH18-2*	Glyma.01G142400.1	283	31.26	6.00	26.98	90.67	0.000	No
*GmGH18-3*	Glyma.02G113600.1	296	31.69	5.18	34.54	79.86	−0.106	Yes
*GmGH18-4*	Glyma.03G254300.1	303	32.59	8.97	38.48	76.90	−0.231	Yes
*GmGH18-5.1*	Glyma.03G256800.1	416	46.84	9.18	50.77	90.05	−0.323	No
*GmGH18-5.2*	Glyma.03G256800.2	418	47.02	9.10	50.37	89.86	−0.325	No
*GmGH18-6*	Glyma.05G075000.1	298	32.64	9.41	38.72	82.21	−0.115	Yes
*GmGH18-7*	Glyma.07G061600.1	289	31.30	6.31	23.17	81.38	−0.068	No
*GmGH18-8*	Glyma.07G061800.1	173	19.46	8.42	32.33	77.28	−0.296	No
*GmGH18-9*	Glyma.08G299700.1	300	32.00	8.08	38.61	86.57	0.024	Yes
*GmGH18-10*	Glyma.08G300300.1	245	25.86	4.87	35.41	87.31	0.106	Yes
*GmGH18-11*	Glyma.09G121000.1	309	34.04	5.18	39.07	79.19	−0.130	Yes
*GmGH18-12*	Glyma.09G126200.1	292	30.88	4.07	33.02	87.57	−0.016	Yes
*GmGH18-13*	Glyma.10G227700.1	304	32.43	7.58	39.30	85.72	0.049	Yes
*GmGH18-14*	Glyma.12G156600.1	298	31.51	5.51	30.94	83.26	−0.050	Yes
*GmGH18-15*	Glyma.13G155800.1	379	41.07	4.78	16.18	87.81	0.141	Yes
*GmGH18-16*	Glyma.13G330800.1	308	34.15	5.31	34.00	83.25	−0.061	Yes
*GmGH18-17*	Glyma.13G330900.1	264	30.11	5.10	42.11	79.32	−0.352	No
*GmGH18-18.1*	Glyma.15G015100.1	624	68.80	8.29	33.35	89.58	−0.072	Yes
*GmGH18-18.2*	Glyma.15G015100.2	820	91.01	6.31	35.59	90.16	−0.141	Yes
*GmGH18-18.3*	Glyma.15G015100.3	624	68.80	8.29	33.35	89.58	−0.072	Yes
*GmGH18-19*	Glyma.15G043300.1	298	34.24	5.67	30.69	80.17	−0.337	No
*GmGH18-20*	Glyma.15G206400.1	762	86.08	6.40	39.94	92.02	−0.167	Yes
*GmGH18-21*	Glyma.15G206800.1	365	40.09	8.92	34.77	73.29	−0.304	Yes
*GmGH18-22.1*	Glyma.16G173000.1	297	31.77	5.01	34.00	87.74	−0.043	Yes
*GmGH18-22.2*	Glyma.16G173000.2	295	31.62	5.01	34.16	88.34	−0.039	Yes
*GmGH18-23*	Glyma.17G076100.1	374	41.25	8.79	33.08	70.99	−0.102	Yes
*GmGH18-24*	Glyma.17G103500.1	377	41.06	9.11	18.08	88.81	0.158	Yes
*GmGH18-25*	Glyma.17G217000.1	384	43.29	6.14	32.33	79.79	−0.228	Yes
*GmGH18-26*	Glyma.18G120200.1	295	31.23	5.87	35.97	85.08	0.095	Yes
*GmGH18-27*	Glyma.18G120700.1	295	31.27	7.50	32.96	85.08	0.045	Yes
*GmGH18-28*	Glyma.18G120800.1	211	22.89	4.36	29.29	89.19	0.149	Yes
*GmGH18-29*	Glyma.18G120900.1	261	27.86	8.14	33.54	85.25	0.026	Yes
*GmGH18-30*	Glyma.19G076200.1	316	34.75	9.42	37.34	77.53	−0.238	Yes
*GmGH18-31*	Glyma.19G255000.1	175	20.03	8.65	45.49	90.34	−0.114	No
*GmGH18-32.1*	Glyma.20G035400.1	800	88.94	7.93	42.27	87.72	−0.170	Yes
*GmGH18-32.2*	Glyma.20G035400.2	798	88.77	7.93	42.35	87.46	−0.178	Yes
*GmGH18-33*	Glyma.20G164600.1	301	32.39	9.34	41.78	84.29	−0.017	Yes
*GmGH18-34*	Glyma.20G164700.1	333	37.03	4.87	32.39	79.97	−0.163	Yes
*GmGH18-35*	Glyma.20G164900.1	299	32.11	4.27	38.89	83.58	−0.092	Yes
*GmGH18-36*	Glyma.U033800.1	302	33.54	5.44	33.17	78.15	−0.118	Yes

## Data Availability

All data generated or analyzed in this study are included in the main text and its Supplementary Materials.

## Supplementary Materials

The following supporting information can be downloaded at: https://www.mdpi.com/article/10.3390/ijms26041649/s1.

## References

[1] Chen W., Jiang X., Yang Q. (2020). Glycoside hydrolase family 18 chitinases: The known and the unknown. Biotechnol. Adv..

[2] Khan R.S., Iqbal A., Bibi A., Khalil I., Ul Islam Z., Jan F., Khalid A., Abdalla A.N., Wadood A. (2024). Plant chitinases: Types, structural classification, antifungal potential and transgenic expression in plants for enhanced disease resistance. Plant Cell Tissue Organ Cult..

[3] Mahajan G., Sharma V., Gupta R. (2024). Chitinase: A potent biocatalyst and its diverse applications. Biocatal. Biotransform..

[4] Drula E., Garron M.L., Dogan S., Lombard V., Henrissat B., Terrapon N. (2022). The carbohydrate-active enzyme database: Functions and literature. Nucleic Acids Res..

[5] Sharma A., Arya S.K., Singh J., Kapoor B., Bhatti J.S., Suttee A., Singh G. (2024). Prospects of chitinase in sustainable farming and modern biotechnology: An update on recent progress and challenges. Biotechnol. Genet. Eng. Rev..

[6] Xie Z.-P., Staehelin C., Wiemken A., Boller T. (1996). Ethylene responsiveness of soybean cultivars characterized by leaf senescence, chitinase induction and nodulation. J. Plant Physiol..

[7] Xie Z.-P., Staehelin C., Wiemken A., Broughton W.J., Müller J., Boller T. (1999). Symbiosis-stimulated chitinase isoenzymes of soybean (*Glycine max* (L.) Merr.). J. Exp. Bot..

[8] Grover A. (2012). Plant chitinases: Genetic diversity and physiological roles. Crit. Rev. Plant Sci..

[9] Meins F., Fritig B., Linthorst H.J., Mikkelsen J.D., Neuhaus J.-M., Ryals J. (1994). Plant chitinase genes. Plant Mol. Biol. Rep..

[10] Neuhaus J.-M., Fritig B., Linthorst H., Meins F., Mikkelsen J., Ryals J. (1996). A revised nomenclature for chitinase genes. Plant Mol. Biol. Rep..

[11] Melchers L.S., Apotheker-de Groot M., van der Knaap J.A., Ponstein A.S., Sela-Buurlage M.B., Bol J.F., Cornelissen B.J., van den Elzen P.J., Linthorst H.J. (1994). A new class of tobacco chitinases homologous to bacterial exo-chitinases displays antifungal activity. Plant J..

[12] Brunner F., Stintzi A., Fritig B., Legrand M. (1998). Substrate specificities of tobacco chitinases. Plant J..

[13] Ohnuma T., Numata T., Osawa T., Mizuhara M., Lampela O., Juffer A.H., Skriver K., Fukamizo T. (2011). A class V chitinase from *Arabidopsis thaliana*: Gene responses, enzymatic properties, and crystallographic analysis. Planta.

[14] Schlöffel M.A., Käsbauer C., Gust A.A. (2019). Interplay of plant glycan hydrolases and LysM proteins in plant—Bacteria interactions. Int. J. Med. Microbiol..

[15] Salzer P., Bonanomi A., Beyer K., Vögeli-Lange R., Aeschbacher R.A., Lange J., Wiemken A., Kim D., Cook D.R., Boller T. (2000). Differential expression of eight chitinase genes in *Medicago truncatula* roots during mycorrhiza formation, nodulation, and pathogen infection. Mol. Plant-Microbe Interact..

[16] Frettinger P., Herrmann S., Lapeyrie F., Oelmüller R., Buscot F. (2006). Differential expression of two class III chitinases in two types of roots of *Quercus robur* during pre-mycorrhizal interactions with *Piloderma croceum*. Mycorrhiza.

[17] Li H.Y., Yang G.D., Shu H.R., Yang Y.T., Ye B.X., Nishida I., Zheng C.C. (2006). Colonization by the arbuscular mycorrhizal fungus *Glomus versiforme* induces a defense response against the root-knot nematode *Meloidogyne incognita* in the grapevine (*Vitis amurensis* Rupr.), which includes transcriptional activation of the class III chitinase gene *VCH3*. Plant Cell Physiol..

[18] Goormachtig S., Lievens S., Van de Velde W., Van Montagu M., Holsters M. (1998). Srchi13, a novel early nodulin from *Sesbania rostrata*, is related to acidic class III chitinases. Plant Cell.

[19] Fortunato A., Santos P., Graça I., Gouveia M.M., Martins S.M., Pinto Ricardo C.P., Pawlowski K., Ribeiro A. (2007). Isolation and characterization of *cgchi3*, a nodule-specific gene from *Casuarina glauca* encoding a class III chitinase. Physiol. Plant..

[20] Tian Y., Liu W., Cai J., Zhang L.Y., Wong K.B., Feddermann N., Boller T., Xie Z.P., Staehelin C. (2013). The nodulation factor hydrolase of *Medicago truncatula*: Characterization of an enzyme specifically cleaving rhizobial nodulation signals. Plant Physiol..

[21] Breakspear A., Liu C., Roy S., Stacey N., Rogers C., Trick M., Morieri G., Mysore K.S., Wen J., Oldroyd G.E. (2014). The root hair “infectome” of *Medicago truncatula* uncovers changes in cell cycle genes and reveals a requirement for Auxin signaling in rhizobial infection. Plant Cell.

[22] Cai J., Zhang L.Y., Liu W., Tian Y., Xiong J.S., Wang Y.H., Li R.J., Li H.M., Wen J., Mysore K.S. (2018). Role of the Nod Factor Hydrolase MtNFH1 in regulating Nod factor levels during rhizobial infection and in mature nodules of *Medicago truncatula*. Plant Cell.

[23] Li R.J., Zhang C.X., Fan S.Y., Wang Y.H., Wen J., Mysore K.S., Xie Z.P., Staehelin C. (2022). The *Medicago truncatula* hydrolase MtCHIT5b degrades Nod factors of *Sinorhizobium meliloti* and cooperates with MtNFH1 to regulate the nodule symbiosis. Front. Plant Sci..

[24] Malolepszy A., Kelly S., Sørensen K.K., James E.K., Kalisch C., Bozsoki Z., Panting M., Andersen S.U., Sato S., Tao K. (2018). A plant chitinase controls cortical infection thread progression and nitrogen-fixing symbiosis. eLife.

[25] Biswas B., Gresshoff P.M. (2014). The role of symbiotic nitrogen fixation in sustainable production of biofuels. Int. J. Mol. Sci..

[26] Lv P., Zhang C., Xie P., Yang X., El-Sheikh M.A., Hefft D.I., Ahmad P., Zhao T., Bhat J.A. (2022). Genome-wide identification and expression analyses of the chitinase gene family in response to white mold and drought stress in soybean (*Glycine max*). Life.

[27] Chen J.Y., Sang H., Chilvers M.I., Wu C.H., Chang H.X. (2024). Characterization of soybean chitinase genes induced by rhizobacteria involved in the defense against *Fusarium oxysporum*. Front. Plant Sci..

[28] Coulson A.F. (1994). A proposed structure for ‘family 18’ chitinases. A possible function for narbonin. FEBS Lett..

[29] Magadum S., Banerjee U., Murugan P., Gangapur D., Ravikesavan R. (2013). Gene duplication as a major force in evolution. J. Genet..

[30] Landis J.B., Soltis D.E., Li Z., Marx H.E., Barker M.S., Tank D.C., Soltis P.S. (2018). Impact of whole-genome duplication events on diversification rates in angiosperms. Am. J. Bot..

[31] Tang H., Bowers J.E., Wang X., Ming R., Alam M., Paterson A.H. (2008). Synteny and collinearity in plant genomes. Science.

[32] Schmutz J., Cannon S.B., Schlueter J., Ma J., Mitros T., Nelson W., Hyten D.L., Song Q., Thelen J.J., Cheng J. (2010). Genome sequence of the palaeopolyploid soybean. Nature.

[33] Cannon S.B., Sterck L., Rombauts S., Sato S., Cheung F., Gouzy J., Wang X., Mudge J., Vasdewani J., Schiex T. (2006). Legume genome evolution viewed through the *Medicago truncatula* and *Lotus japonicus* genomes. Proc. Natl. Acad. Sci. USA.

[34] Takenaka Y., Nakano S., Tamoi M., Sakuda S., Fukamizo T. (2009). Chitinase gene expression in response to environmental stresses in *Arabidopsis thaliana*: Chitinase inhibitor allosamidin enhances stress tolerance. Biosci. Biotechnol. Biochem..

[35] Vaghela B., Vashi R., Rajput K., Joshi R. (2022). Plant chitinases and their role in plant defense: A comprehensive review. Enzym. Microb. Technol..

[36] De Jong A.J., Cordewener J., Lo Schiavo F., Terzi M., Vandekerckhove J., Van Kammen A., De Vries S.C. (1992). A carrot somatic embryo mutant is rescued by chitinase. Plant Cell.

[37] Perret X., Staehelin C., Broughton W.J. (2000). Molecular basis of symbiotic promiscuity. Microbiol. Mol. Biol. Rev..

[38] Heidstra R., Geurts R., Franssen H., Spaink H.P., Van Kammen A., Bisseling T. (1994). Root hair deformation activity of nodulation factors and their fate on *Vicia sativa*. Plant Physiol..

[39] Staehelin C., Schultze M., Kondorosi E., Kondorosi A. (1995). Lipo-chitooligosaccharide nodulation signals from *Rhizobium meliloti* induce their rapid degradation by the host plant alfalfa. Plant Physiol..

[40] Ovtsyna A.O., Dolgikh E.A., Kilanova A.S., Tsyganov V.E., Borisov A.Y., Tikhonovich I.A., Staehelin C. (2005). Nod factors induce Nod factor cleaving enzymes in pea roots. Genetic and pharmacological approaches indicate different activation mechanisms. Plant Physiol..

[41] Chen W., Wang D., Ke S., Cao Y., Xiang W., Guo X., Yang Q. (2024). A soybean cyst nematode suppresses microbial plant symbionts using a lipochitooligosaccharide-hydrolysing enzyme. Nat. Microbiol..

[42] Tang H., Krishnakumar V., Bidwell S., Rosen B., Chan A., Zhou S., Gentzbittel L., Childs K.L., Yandell M., Gundlach H. (2014). An improved genome release (version Mt4.0) for the model legume *Medicago truncatula*. BMC Genom..

[43] Li H., Jiang F., Wu P., Wang K., Cao Y. (2020). A high-quality genome sequence of model legume *Lotus japonicus* (MG-20) provides insights into the evolution of root nodule symbiosis. Genes.

[44] Chen C., Wu Y., Li J., Wang X., Zeng Z., Xu J., Liu Y., Feng J., Chen H., He Y. (2023). TBtools-II: A “one for all, all for one” bioinformatics platform for biological big-data mining. Mol. Plant.

[45] Whelan S., Goldman N. (2001). A general empirical model of protein evolution derived from multiple protein families using a maximum-likelihood approach. Mol. Biol. Evol..

[46] Kumar S., Stecher G., Tamura K. (2016). MEGA7: Molecular evolutionary genetics analysis version 7.0 for bigger datasets. Mol. Biol. Evol..

[47] Bailey T.L., Boden M., Buske F.A., Frith M., Grant C.E., Clementi L., Ren J., Li W.W., Noble W.S. (2009). MEME SUITE: Tools for motif discovery and searching. Nucleic Acids Res..

[48] Wang J., Chitsaz F., Derbyshire M.K., Gonzales N.R., Gwadz M., Lu S., Marchler G.H., Song J.S., Thanki N., Yamashita R.A. (2023). The conserved domain database in 2023. Nucleic Acids Res..

[49] Gasteiger E., Hoogland C., Gattiker A., Duvaud S.E., Wilkins M.R., Appel R.D., Bairoch A. (2005). Protein Identification and Analysis Tools on the ExPASy Server.

[50] Teufel F., Almagro Armenteros J.J., Johansen A.R., Gíslason M.H., Pihl S.I., Tsirigos K.D., Winther O., Brunak S., von Heijne G., Nielsen H. (2022). SignalP 6.0 predicts all five types of signal peptides using protein language models. Nat. Biotechnol..

[51] Wang Y., Tang H., Debarry J.D., Tan X., Li J., Wang X., Lee T.H., Jin H., Marler B., Guo H. (2012). MCScanX: A toolkit for detection and evolutionary analysis of gene synteny and collinearity. Nucleic Acids Res..

[52] Lescot M., Déhais P., Thijs G., Marchal K., Moreau Y., Van de Peer Y., Rouzé P., Rombauts S. (2002). PlantCARE, a database of plant cis-acting regulatory elements and a portal to tools for in silico analysis of promoter sequences. Nucleic Acids Res..

[53] Chow C.N., Yang C.W., Wu N.Y., Wang H.T., Tseng K.C., Chiu Y.H., Lee T.Y., Chang W.C. (2024). PlantPAN 4.0: Updated database for identifying conserved non-coding sequences and exploring dynamic transcriptional regulation in plant promoters. Nucleic Acids Res..

[54] Kelly S., Mun T., Stougaard J., Ben C., Andersen S.U. (2018). Distinct *Lotus japonicus* transcriptomic responses to a spectrum of bacteria ranging from symbiotic to pathogenic. Front. Plant Sci..

