# Supplementary material for: Genome-Wide Identification and Expression Analyses of Glycoside Hydrolase Family 18 Genes During Nodule Symbiosis in Glycine max
Source: Int J Mol Sci. 2025 Feb 14;26(4):1649. doi: 10.3390/ijms26041649 (PMC11855358; doi:10.3390/ijms26041649)
Supplement: Supplementary file 1 [file ijms-26-01649-s001.zip › ijms-3393647-supplementary.pdf]

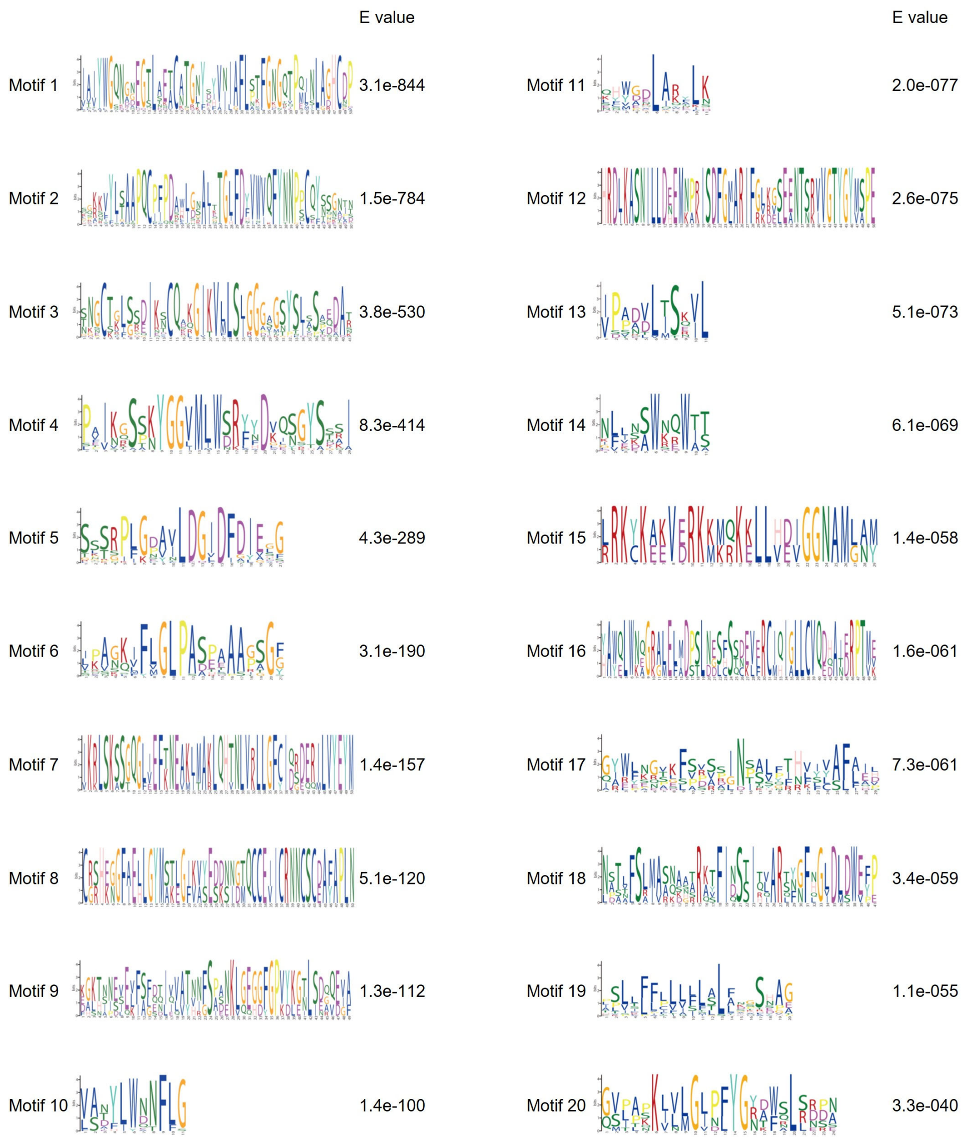

Figure S1. Sequence logos of the conserved motifs. Motif analysis of soybean GH18 proteins were performed using MEME Suite 5.5.5 online service (https://meme-suite.org/). The sequences of top 10 motifs are shown with E value labeled on the right.

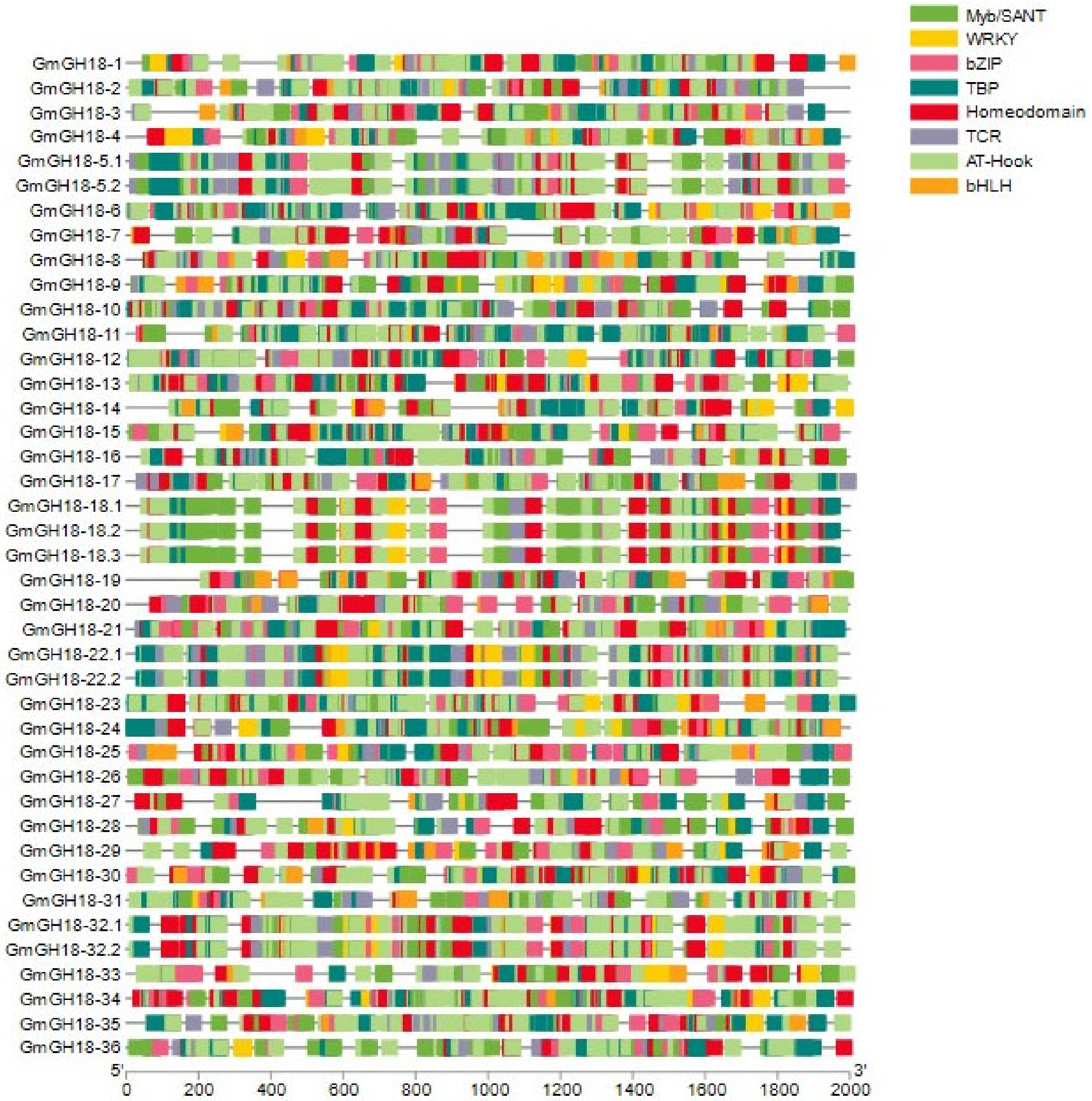

Figure S2. Analysis of the TF binding sites in the promoter regions of *G. max GH18* genes. 41 *GH18* gene promoter sequences (-2000 bp upstream of ATG) are obtained from *G. max* Wm82 genome (a2.v1). The gene symbols are listed on the left and the nucleotide positions are labeled at the bottom. AT-Hook, TBP, Homeodomain, Myb/SANT, WRKY, bHLH, bZIP and TCR were identified.

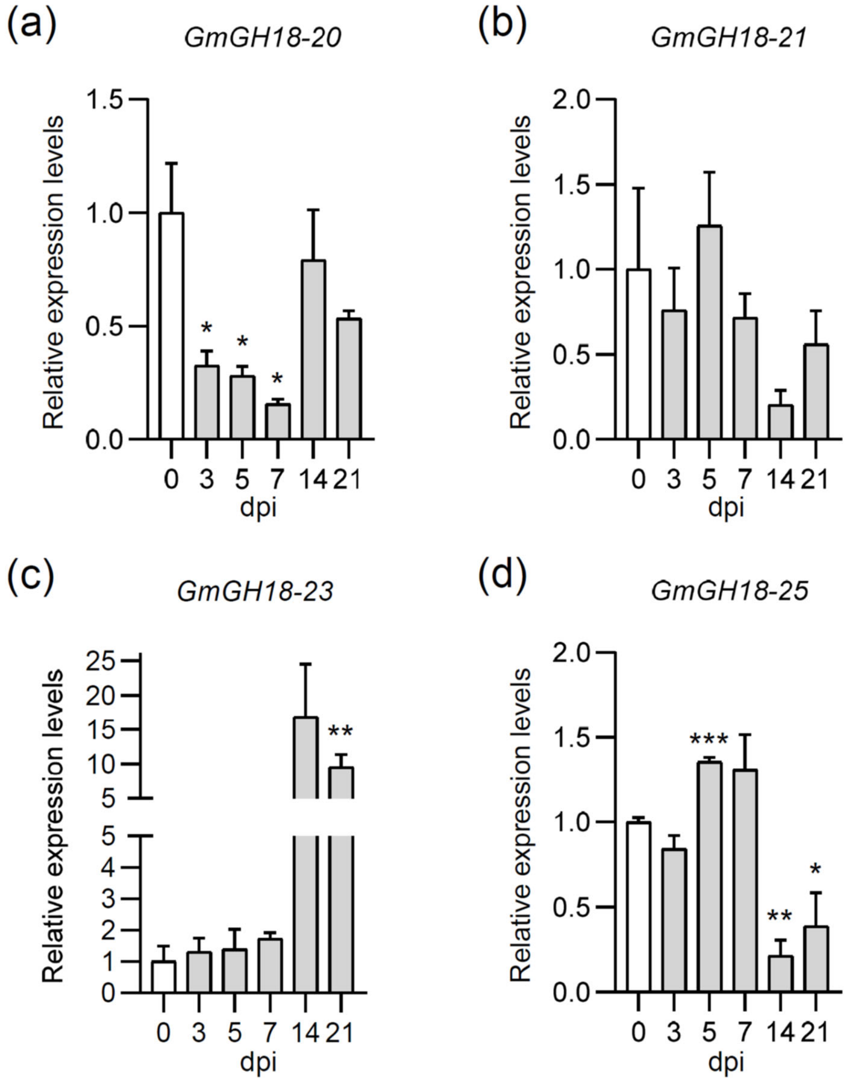

Figure S3. Four soybean *GH18* gene transcript levels were measured under symbiotic conditions. Seedlings of *G. max* Wm82 were inoculated with *B. japonium* USDA110. Roots (within 7 days post inoculation) and nodules (14 and 21 dpi) were harvested at corresponding time points for RNA extraction. The transcript levels of *GmGH18-20* (a), *GmGH18-21* (b), *GmGH18-23* (c) and *GmGH18-25* (d) were determined by RT-qPCR ($n = 3$). Data indicate means ± SE of normalized expression values (mean value of control set to one). The asterisks indicate significantly increased (or decreased) expression compared to control roots without rhizobial inoculation (Student's *t*-test; * $P < 0.05$, ** $P < 0.01$, ***, $P < 0.001$).

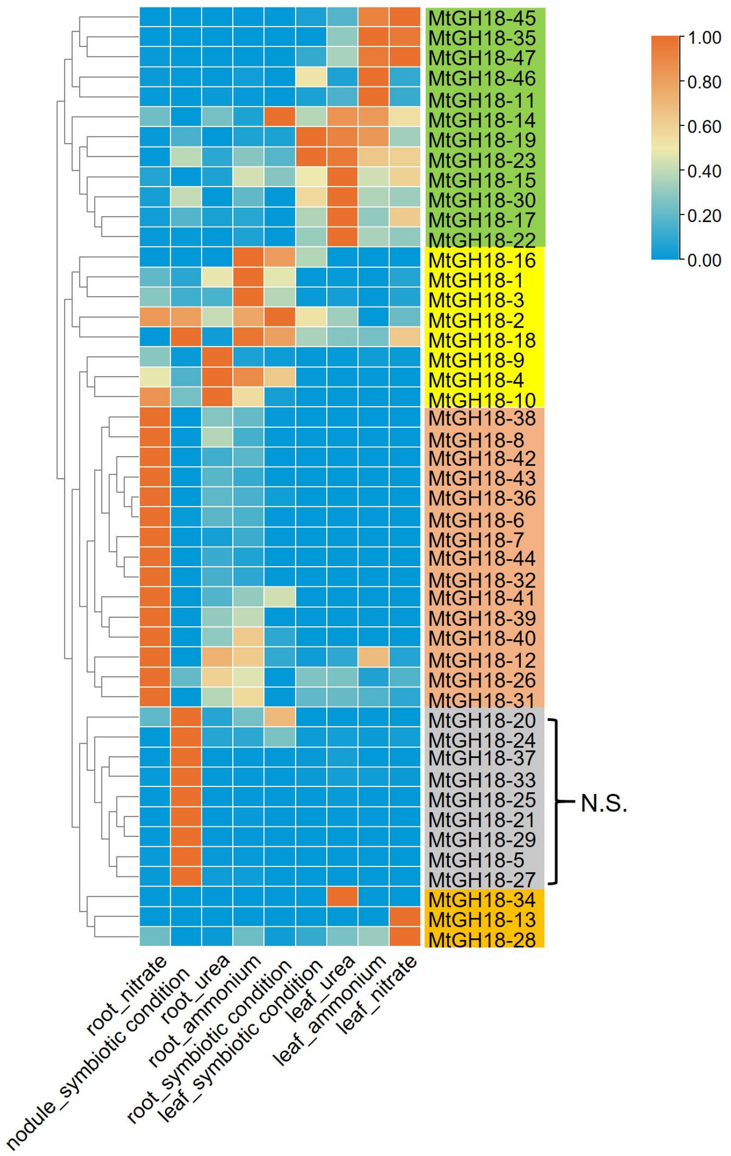

Figure S4. Expression profile of Medicago *GH18* genes under nitrogen acquisition related conditions. *M. truncatula* plants were treated with nitrate, urea, ammonium or inoculated with rhizobia. Roots, nodules and leaves were harvested for RNA extraction. RNA sequencing data normalized in row scale was displayed in the heatmap. N.S., nodulation specific.

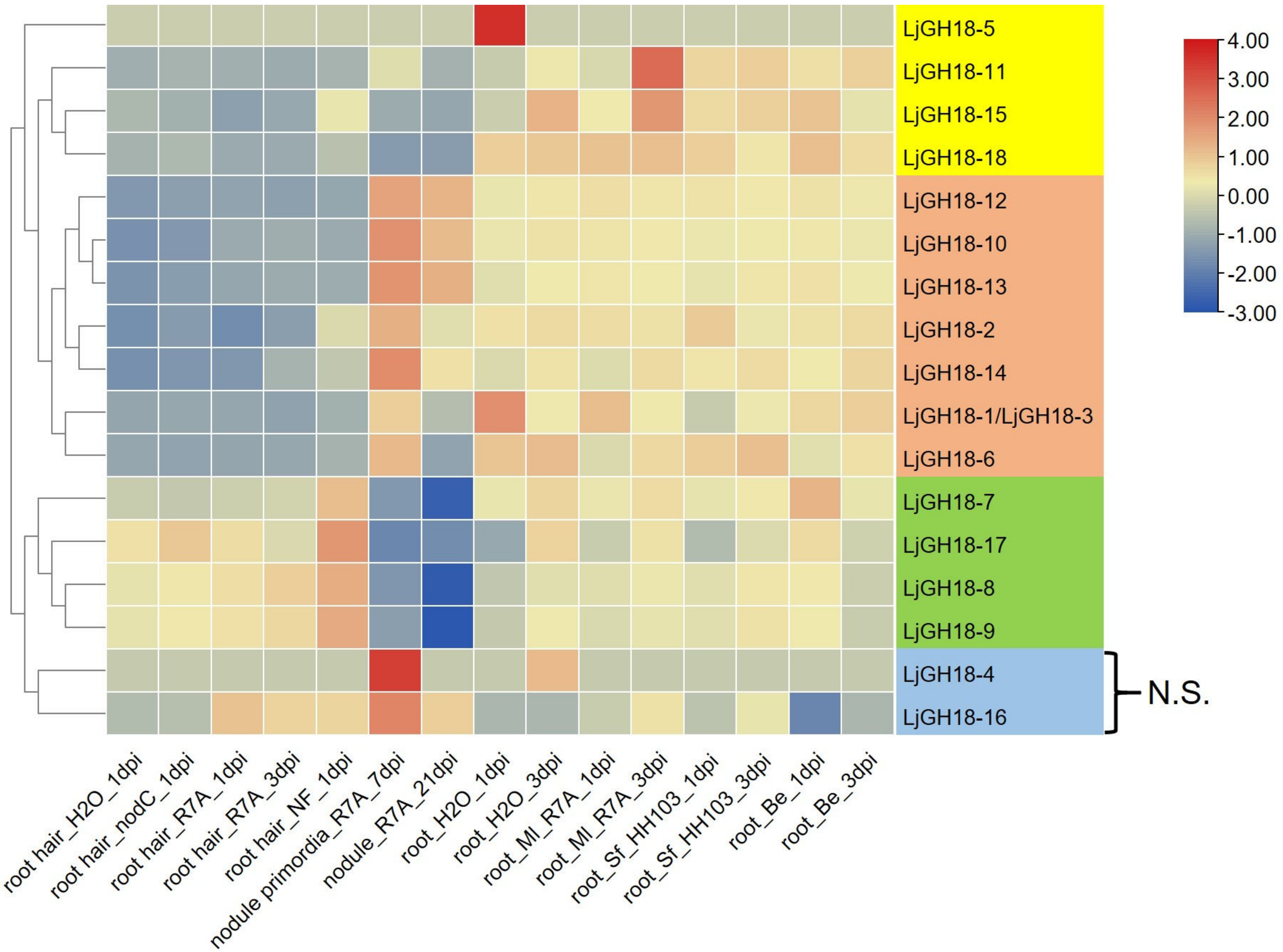

Figure S5. Expression profile of Lotus *GH18* genes under symbiotic conditions. *L. japonicus* ecotype Gifu seedlings were treated with *Mesorhizobium loti* R7A, the NF-deficient strain R7A*nodC*, purified NF, *Sinorhizobium fredii* HH103 and *Bradyrhizobium elkanii* USDA61, respectively. Root hairs or roots were harvested at different time points for RNA extraction [54]. The RNA sequencing data normalized in row scale was displayed in the heatmap. N.S., nodulation specific.

Table S1. *GH18* gene ID list of corresponding gene symbol in Medicago and Lotus.

Gene symbol	Gene ID	Gene symbol	Gene ID	Gene symbol	Gene ID
MtGH18-1	Medtr1g013150.1	MtGH18-22	Medtr4g117020.1	MtGH18-44	Medtr0062s0170.1
MtGH18-2	Medtr1g099290.1	MtGH18-23	Medtr4g117030.1	MtGH18-45	Medtr0263s0020.1
MtGH18-3	Medtr1g099310.1	MtGH18-24	Medtr4g117040.1	MtGH18-46	Medtr0443s0040.1
MtGH18-4	Medtr1g099320.1	MtGH18-25	Medtr4g117800.1	MtGH18-47	Medtr1457s0010.1
MtGH18-5	Medtr1g099350.1	MtGH18-26	Medtr5g025480.1	LjGH18-1	Lj1g0009167.1
MtGH18-6	Medtr2g036240.1	MtGH18-27	Medtr5g043550.1	LjGH18-2	Lj1g0009750.1
MtGH18-7	Medtr2g036260.1	MtGH18-28	Medtr6g023340.1	LjGH18-3	Lj1g0013482.1
MtGH18-8	Medtr2g036290.1	MtGH18-29	Medtr6g079630.1	LjGH18-4	Lj2g0000013.1
MtGH18-9	Medtr2g040650.1	MtGH18-30	Medtr7g116350.1	LjGH18-5	Lj2g0018868.1
MtGH18-10	Medtr2g040690.1	MtGH18-31	Medtr7g116850.1	LjGH18-6	Lj3g0000874.1
MtGH18-11	Medtr2g040740.1	MtGH18-32	Medtr8g046730.1	LjGH18-7	Lj3g0002176.1
MtGH18-12	Medtr2g040830.1	MtGH18-33	Medtr8g055940.1	LjGH18-8	Lj3g0026062.1
MtGH18-13	Medtr2g040870.1	MtGH18-34	Medtr8g467650.1	LjGH18-9	Lj5g0001195.1
MtGH18-14	Medtr2g040890.1	MtGH18-35	Medtr0002s0040.1	LjGH18-10	Lj5g0003799.1
MtGH18-15	Medtr2g096030.1	MtGH18-36	Medtr0002s1060.1	LjGH18-11	Lj5g0004169.1
MtGH18-16	Medtr2g102020.1	MtGH18-37	Medtr0027s0260.1	LjGH18-12	Lj5g0006187.1
MtGH18-17	Medtr3g110280.1	MtGH18-38	Medtr0062s0020.1	LjGH18-13	Lj5g0009602.1
MtGH18-18.1	Medtr3g110320.1	MtGH18-39	Medtr0062s0040.1	LjGH18-14	Lj5g0018714.1
MtGH18-18.2	Medtr3g110320.2	MtGH18-40	Medtr0062s0060.1	LjGH18-15	Lj6g0000028.1
MtGH18-19	Medtr4g116920.1	MtGH18-41	Medtr0062s0090.1	LjGH18-16	Lj4g0016455.1
MtGH18-20	Medtr4g116990.1	MtGH18-42	Medtr0062s0110.1	LjGH18-17	Lj4g0024525.1
MtGH18-21	Medtr4g117000.1	MtGH18-43	Medtr0062s0130.1	LjGH18-18	Lj4g0025435.1

Table S2. 19 pairs of fragment copy genes identified in soybean *GH18* gene family.

NO.	Gene symbol	Gene ID	Gene symbol	Gene ID
1	GmGH18-1	Glyma.01G055200.1	GmGH18-3	Glyma.02G113600.1
2	GmGH18-2	Glyma.01G142400.1	-	Glyma.03G030500.1
3	GmGH18-4	Glyma.03G254300.1	-	Glyma.19G251900.1
4	GmGH18-5	Glyma.03G256800.1	GmGH18-31	Glyma.19G255000.1
5	GmGH18-6	Glyma.05G075000.1	GmGH18-30	Glyma.19G076200.1
6	-	Glyma.07G005700.1	GmGH18-18	Glyma.15G015100.1
7	GmGH18-12	Glyma.09G126200.1	GmGH18-13	Glyma.10G227700.1
8	GmGH18-12	Glyma.09G126200.1	GmGH18-22	Glyma.16G173000.1
9	GmGH18-12	Glyma.09G126200.1	GmGH18-33	Glyma.20G164600.1
10	GmGH18-13	Glyma.10G227700.1	GmGH18-22	Glyma.16G173000.1
11	GmGH18-13	Glyma.10G227700.1	GmGH18-33	Glyma.20G164600.1
12	GmGH18-15	Glyma.13G155800.1	GmGH18-20	Glyma.15G206400.1
13	GmGH18-17	Glyma.13G330900.1	GmGH18-19	Glyma.15G043300.1
14	-	Glyma.13G358900.1	GmGH18-18	Glyma.15G015100.1
15	GmGH18-15	Glyma.13G155800.1	GmGH18-24	Glyma.17G103500.1
16	GmGH18-15	Glyma.13G155800.1	GmGH18-23	Glyma.17G076100.1
17	GmGH18-16	Glyma.13G330800.1	GmGH18-36	Glyma.U033800.1
18	-	Glyma.14G110700.1	GmGH18-25	Glyma.17G217000.1
19	GmGH18-22	Glyma.16G173000.1	GmGH18-33	Glyma.20G164600.1

Table S3. 17 pairs of fragment copy *GH18* genes identified between soybean and Medicago.

NO.	Gene symbol	Gene ID	Gene symbol	Gene ID
1	GmGH18-1	Glyma.01G055200.1	MtGH18-27	Medtr5g043550.1
2	GmGH18-3	Glyma.02G113600.1	MtGH18-27	Medtr5g043550.1
3	GmGH18-4	Glyma.03G254300.1	MtGH18-31	Medtr7g116850.1
4	GmGH18-6	Glyma.05G075000.1	MtGH18-28	Medtr6g023340.1
5	GmGH18-9	Glyma.08G299700.1	MtGH18-34	Medtr8g467650.1
6	GmGH18-12	Glyma.09G126200.1	MtGH18-2	Medtr1g099290.1
7	GmGH18-13	Glyma.10G227700.1	MtGH18-3	Medtr1g099310.1
8	GmGH18-16	Glyma.13G330800.1	MtGH18-15	Medtr2g096030.1
9	GmGH18-15	Glyma.13G155800.1	MtGH18-20	Medtr4g116990.1
10	GmGH18-19	Glyma.15G043300.1	MtGH18-15	Medtr2g096030.1
11	GmGH18-22	Glyma.16G173000.1	MtGH18-2	Medtr1g099290.1
12	GmGH18-25	Glyma.17G217000.1	MtGH18-1	Medtr1g013150.1
13	GmGH18-24	Glyma.17G103500.1	MtGH18-20	Medtr4g116990.1
14	GmGH18-26	Glyma.18G120200.1	MtGH18-33	Medtr8g055940.1
15	GmGH18-30	Glyma.19G076200.1	MtGH18-28	Medtr6g023340.1
16	GmGH18-33	Glyma.20G164600.1	MtGH18-3	Medtr1g099310.1
17	GmGH18-35	Glyma.20G164900.1	MtGH18-2	Medtr1g099290.1

Table S4. 23 pairs of fragment copy *GH18* genes identified between soybean and Lotus.

NO.	Gene symbol	Gene ID	Gene symbol	Gene ID
1	GmGH18-1	Glyma.01G055200.1	LjGH18-4	Lj2g0000013.1
2	GmGH18-3	Glyma.02G113600.1	LjGH18-4	Lj2g0000013.1
3	GmGH18-7	Glyma.07G061600.1	LjGH18-3	Lj1g0013482.1
4	GmGH18-13	Glyma.10G227700.1	LjGH18-9	Lj5g0001195.1
5	GmGH18-16	Glyma.13G330800.1	LjGH18-6	Lj3g0000874.1
6	GmGH18-16	Glyma.13G330800.1	-	Lj3g0018806.1
7	GmGH18-15	Glyma.13G155800.1	LjGH18-16	Lj4g0016455.1
8	GmGH18-15	Glyma.13G155800.1	LjGH18-15	Lj6g0000028.1
9	GmGH18-19	Glyma.15G043300.1	-	Lj3g0018806.1
10	GmGH18-19	Glyma.15G043300.1	LjGH18-6	Lj3g0000874.1
11	GmGH18-18	Glyma.15G015100.1	-	Lj3g0023389.1
12	GmGH18-21	Glyma.15G206800.1	LjGH18-17	Lj4g0024525.1
13	GmGH18-21	Glyma.15G206800.1	LjGH18-15	Lj6g0000028.1
14	GmGH18-22	Glyma.16G173000.1	LjGH18-9	Lj5g0001195.1
15	GmGH18-23	Glyma.17G076100.1	LjGH18-17	Lj4g0024525.1
16	GmGH18-24	Glyma.17G103500.1	LjGH18-16	Lj4g0016455.1
17	GmGH18-25	Glyma.17G217000.1	LjGH18-16	Lj4g0016455.1
18	GmGH18-25	Glyma.17G217000.1	LjGH18-13	Lj5g0009602.1
19	GmGH18-23	Glyma.17G076100.1	LjGH18-15	Lj6g0000028.1
20	GmGH18-24	Glyma.17G103500.1	LjGH18-15	Lj6g0000028.1
21	GmGH18-25	Glyma.17G217000.1	LjGH18-15	Lj6g0000028.1
22	GmGH18-33	Glyma.20G164600.1	LjGH18-9	Lj5g0001195.1
23	GmGH18-34	Glyma.20G164700.1	LjGH18-14	Lj5g0018714.1

Table S5. 8 pairs of fragment copy *GH18* genes identified between Medicago and Lotus.

NO.	Gene symbol	Gene ID	Gene symbol	Gene ID
1	MtGH18-27	Medtr5g043550.1	LjGH18-4	Lj2g0000013.1
2	MtGH18-22	Medtr4g117020.1	LjGH18-18	Lj4g0025435.1
3	MtGH18-1	Medtr1g013150.1	LjGH18-16	Lj4g0016455.1
4	MtGH18-1	Medtr1g013150.1	LjGH18-13	Lj5g0009602.1
5	MtGH18-2	Medtr1g099290.1	LjGH18-9	Lj5g0001195.1
6	MtGH18-16	Medtr2g102020.1	LjGH18-8	Lj3g0026062.1
7	MtGH18-15	Medtr2g096030.1	-	Lj3g0018806.1
8	MtGH18-15	Medtr2g096030.1	LjGH18-6	Lj3g0000874.1

Table S6. The list of primers used in this study.

Primer name	Sequence
qRT-*Actin*-F	ACCCAGCAGCATGAAGATCA
qRT-*Actin*-R	CACATCTGCTGGAAGGTGCT
qRT-*GmGH18-15*-F	GTCGGGTACGATGACCCGAAAACG
qRT-*GmGH18-15*-R	GCATTCAGAGCTTGTGTTGAG
qRT-*GmGH18-20*-F	ATACTCAACCAAATCCGATGTG
qRT-*GmGH18-20*-R	TCCATAGCTCATAAGCATACTCC
qRT-*GmGH18-21*-F	ATGGTTACAAGTGGAGTTTGTCAG
qRT-*GmGH18-21*-R	GAGAAGCGGCTTGAGAAAGAG
qRT-*GmGH18-23*-F	AACCGTGGATTGTTTGATAGAG
qRT-*GmGH18-23*-R	AGCTGCTTGAGAAAGAACCC
qRT-*GmGH18-24*-F	AGGTGCGAAAGTTGTATATGACAG
qRT-*GmGH18-24*-R	GCATTCAGAGCTTGTGTTGAG
qRT-*GmGH18-25*-F	CACGGATACAGATGGTACTATGG
qRT-*GmGH18-25*-R	TGCATTTGAAGCTTGTCTTGAG
qRT-*GmGH18-33*-F	CCTCTTCTCCTTCCCACTCTC
qRT-*GmGH18-33*-R	CCACCTATTGAAAGCAACACCT
